# Advanced Glycation End Products Enhance Biofilm Formation by Promoting Extracellular DNA Release Through *sigB* Upregulation in *Staphylococcus aureus*

**DOI:** 10.3389/fmicb.2020.01479

**Published:** 2020-07-14

**Authors:** Xiaoying Xie, Xiaoqiang Liu, Yanling Li, Ling Luo, Wenchang Yuan, Baiji Chen, Guoyan Liang, Rui Shen, Hongyu Li, Songyin Huang, Chaohui Duan

**Affiliations:** ^1^Department of Clinical Laboratory, Sun Yat-sen Memorial Hospital, Sun Yat-sen University, Guangzhou, China; ^2^Guangdong Provincial Key Laboratory of Malignant Tumor Epigenetics and Gene Regulation, Sun Yat-sen Memorial Hospital, Sun Yat-sen University, Guangzhou, China; ^3^Department of Blood Transfusion, Sun Yat-sen Memorial Hospital, Sun Yat-sen University, Guangzhou, China; ^4^Department of Clinical Laboratory, The Fifth Affiliated Hospital of Guangzhou Medical University, Guangzhou, China

**Keywords:** diabetic foot infection, advanced glycation end products, *Staphylococcus aureus*, biofilm formation, *sigB*, eDNA

## Abstract

Bacterial biofilms do serious harm to the diabetic foot ulcer (DFU) because they play a crucial role in infection invasion and spread. *Staphylococcus aureus*, the predominant Gram-positive bacteria in diabetic foot infection (DFI), is often associated with colonization and biofilm formation. Through biofilm formation tests *in vitro*, we observed that *S. aureus* bacteria isolated from DFU wounds were more prone to form biofilms than those from non-diabetic patients, while there was no difference in blood sugar between the biofilm (+) diabetics (DB+) and biofilm (-) diabetics (DB-). Furthermore, we found that advanced glycation end products (AGEs) promoted the biofilm formation of *S. aureus* in clinical isolates and laboratory strains *in vitro*, including a methicillin-resistant strain. Analysis of biofilm components demonstrated that the biofilms formed mainly by increasing extracellular DNA (eDNA) release; remarkably, the *S. aureus* global regulator *sigB* was upregulated, and its downstream factor *lrgA* was downregulated after AGE treatments. Mechanism studies using a *sigB*-deleted mutant (Newman-Δ*sigB*) confirmed that AGEs decreased expression of *lrgA* via induction of *sigB*, which is responsible for eDNA release and is a required component for *S. aureus* biofilm development. In conclusion, the present study suggests that AGEs promote *S. aureus* biofilm formation via an eDNA-dependent pathway by regulating *sigB*. The data generated by this study will provide experimental proof and theoretical support to improve DFU infection healing.

## Introduction

Foot infection is a frequent (40–80%) complication of diabetes (Jiang et al., 2015), the most common cause of diabetes-related lower limb amputation and the leading cause of morbidity in diabetes patients (Wukich et al., 2013; Troisi et al., 2016; Sangeorzan, 2018). The unique host environment of diabetes affects the virulence and antibiotic resistance traits of pathogens, and diabetic foot infection (DFI) in particular is difficult to control, easily spread, and responds poorly to antibiotic therapy. *Staphylococcus aureus* is a predominant pathogen in DFI (Xie et al., 2017) and is an opportunistic pathogen that produces a variety of virulence factors and builds notorious biofilms (Archer et al., 2011; McCarthy et al., 2015). Recent studies showed that diabetic patients were more likely to colonize and infect with *S. aureus*, even in diabetic patients without skin lesions (Mottola et al., 2016), but the precise mechanism is currently unclear.

Biofilms are a conglomerate of bacterial cells, proteins, and DNA intercalated in polysaccharide intercellular adhesin (PIA) and play an important role in bacterial colonization and persistent infections in chronic wounds (Donlan and Costerton, 2002). Exactly how biofilms impair the healing process of wounds is not clear, but general consensus suggests that the persistence of biofilms on wounds can delay healing for multiple reasons. These include induction of chronic inflammation caused by host immune responses (Parsek and Singh, 2003), which act as a mechanical barrier to antibodies (de Oliveira et al., 2016), and inhibition of wound healing by preventing keratinocytes and fibroblasts from migrating to the wound bed (Dunyach-Remy et al., 2016).

The biofilm formation is a requisite skill of bacteria for evolution with their respective hosts. Biofilm generation, maturation, and dissociation phenotype depend on a multitude of environmental signals. The host factors, such as different nutrients, pH, and temperature, affect this sessile growth. Compared with other chronic wounds, diabetic wounds are characteristic of hyperglycemia and a series of hyperglycemia−related factors, such as advanced glycation end products (AGEs) (Mitsuhashi et al., 1993). AGEs are formed by Maillard reaction, which takes place between amine-group compounds (proteins, lipids, and nucleic acids) and carboxides (reducing sugar groups) irreversibly. Unlike blood glucose, AGEs can continuously accumulate in biological tissues once formed, even if blood sugar is controlled to a normal level. AGEs have been considered as a more important pathophysiological mechanism for the development of diabetic complications (Vlassara and Uribarri, 2014; Zhang et al., 2016). It is indicated that AGEs play a role in the pathogenesis of impaired diabetic wound healing.

In order to better understand the exact mechanism and characteristics of *S. aureus* biofilm formation in DFI, we investigated the biofilm formation ability of *S. aureus* strains isolated from the skin wounds diabetic and non-diabetic patients with acute or chronic wound infection. To elucidate the effect of host on biofilm formation, we analyzed some clinical indices in the diabetic chronic wound group. Interestingly, there were no significant differences in blood glucose between DB+ and DB- group, which was identified as a biofilm inducer *in vitro* (Waldrop et al., 2014; Kyoui et al., 2016). As AGEs are more stable in diabetic wound tissues that can affect the bacteria more directly and consciously *in vivo*, we supposed AGEs could promote the biofilm formation ability of *S. aureus.* A series of experiments were further carried out to investigate this hypothesis *in vitro*, and we found AGEs indeed promoted the biofilm formation of *S. aureus*, mainly by increasing extracellular DNA (eDNA) release. Global regulator Sigma factor B (σ B), encoded by *sigB* gene, played an important role in the promotion of biofilm by AGEs through the downstream factor *lrg* operons. Taken together, our findings raised the possibility that AGEs, an important diabetic host factor overlooked in previous DFI research, affect the biofilm formation ability of *S. aureus*, which in turn increases *S. aureus* colonization and infection in diabetes.

## Materials and Methods

### Ethics Statement

Ethics approval for this study was granted by the Ethics Committee of Sun Yat-sen Memorial Hospital (Reference number: SYSEC-KY-KS-2020-003, Supplementary Data Sheet S1).

### Patients and Sample Collection

A hospital-based retrospective study of 131 inpatients (66 male and 65 female) with skin wounds infected with *S. aureus* in the Department of Endocrinology and Metabolism and Department of Dermatology of Sun Yat-sen Memorial Hospital between 1 January 2014 and 31 December 2017, was carried out. The subjects included 69 diabetic patients with chronic skin wound (wound duration > 4 weeks) (groups A and B), 35 non-diabetic patients with chronic skin infection (wound duration > 4 weeks) (group C), and 31 non-diabetic patients with acute skin infection (wound duration < 2 weeks) (group D). Among the 69 diabetic patients, 34 cases that presented with DFI were classified as group A; the other 31 cases that presented with chronic skin infection in other parts were classified as group B. Clinical diagnosis of infection was defined by the presence of at least two of the following indicators: local swelling or indurations, >0.5 cm of erythema around the wound, local tenderness or pain, local warmth, and purulent discharge (Lipsky et al., 2013; Hinchliffe et al., 2016). A total of 131 complete surveys were obtained, including laboratory indices such as fasted blood glucose (FBS), HbA1c, total glyceride (TG), total cholesterol (TC), and low-density lipoprotein cholesterol (LDL-C).

Specimens were collected and sent to the microbiology laboratory within 48 h after admission. Samples were collected by swabbing each wound after cleansing using 0.9% sterile saline and debrided as in our previous studies (Xie et al., 2017). Each wound was rotated using sufficient pressure over a 1-cm^2^ area for 5 s with sterilized cotton swab (Rondas et al., 2013), and then, specimens were placed into sterile transport containers and sent to the microbiology laboratory within 30 min. To avoid sample duplication, isolates from the same individual were excluded. All isolates were identified as *S. aureus* using the VITEK^®^ 2 microbial identification system (bioMérieux, Marcy l’Etoile, France), according to the manufacturer’s instructions.

The clinical and demographic characteristics of the 131 patients are shown in Table 1.

**TABLE 1:** Demographic and clinical characteristics of 131 patients.

**Group**	**Case (*n*)**	**Age (years)**	**Gender (male/female)**	**DM duration (years)**	**FBS (mmol/L)**	**HbA1c (%)**	**TG (mmol/L)**	**TC (mmol/L)**	**LDL-C (mmol/L)**	**Percentage of BF(+)**
A	34	55.5 ± 9.4	15/19	5.3 ± 3.9	9.3 ± 4.6	8.2 ± 2.7	4.5 ± 1.2	5.3 ± 2.1	4.0 ± 0.6	58.8%
B	31	50.3 ± 8.3	20/11	6.7 ± 4.2	9.8 ± 3.6	8.8 ± 2.2	3.9 ± 1.3	5.5 ± 1.5	3.8 ± 0.6	48.4%
C	35	46.7 ± 12.3	18/17	0	4.3 ± 1.3^a^	3.1 ± 1.1^a^	1.5 ± 0.9^a^	5.0 ± 1.4	2.5 ± 0.7^a^	28.6%^a^
D	31	52.5 ± 14.6	13/18	0	4.5 ± 0.8^a^	2.9 ± 1.2^a^	2.1 ± 1.4^a^	3.8 ± 1.2^b^	2.8 ± 0.5^a^	32.3%^a^

*DM, diabetes mellitus; FBS, fasting blood glucose; TC, total cholesterol; TG, total glyceride; LDL-C, LDL cholesterol; BF, biofilm; ^*a*^compared with Group A and B, *P* < 0.05; ^*b*^compared with groups A–C, *P* < 0.05.*

### Bacteria and Reagents

*Staphylococcus aureus* ATCC 29213 and ATCC 25923 were obtained from the American Type Culture Collection (Manassas, VA, United States). *S. aureus* NCTC8325 was obtained from the National Collection of Type Cultures (Beijing, China). *S. aureus* strain Newman and a *sigB-*deleted mutant (Newman-Δ*sigB*) were kindly provided by Professor Wenchang Yuan (Guangzhou Medical University, China) (Liu et al., 2018). All the strains above were methicillin-susceptible *S. aureus* (MSSA). *S. aureus* strain SA03 was isolated from a DFI patient in this study and had been typed as MRSA-t437-IV by multilocus sequence typing (MLST) and spa typing. AGE-BSA (ab51995) was obtained from Abcam (Cambridge, MA, United States). Bovine serum albumin (BSA) was obtained from Sigma–Aldrich (St. Louis, MO, United States). LIVE/DEAD BacLight Bacterial Viability kits were obtained from Thermo Fisher Scientific (Waltham, MA, United States). DNase I, sodium metaperiodate, and proteinase K were obtained from Sigma–Aldrich (Darmstadt, Germany). Trizol Reagent^®^ were obtained from Invitrogen (Carlsbad, CA, United States). PrimeScript TM RT Master Mix and SYBR Premix Ex Taq (Perfect Real Time) were obtained from Takara (Tokyo, Japan).

### Growth Curve of Planktonic *S. aureus*

*In vitro*, the growth curve was used to evaluate the effect of AGEs on the growth of *S. aureus* through measuring the optical absorbance (OD630) of the bacteria cultured in trypsin soybean broth (TSB). OD630 was measured every hour for 24 h. To avoid the possible matrix effects of BSA, we used the same concentration of BSA as a negative control of AGE-BSA.

### Quantification of Biofilms

The biofilm formation ability of the *S. aureus* isolates was evaluated with crystal violet (CV) staining (Stepanovic et al., 2000). After overnight culture in TSB at 37°C with shaking at 250 r/min, isolates were adjusted to 0.5 McFarland with 0.85% NaCl and bacterial concentration was 1–1.5 × 10^8^ CFU/ml. Each suspension was diluted with TSB in the ratio of 1:20, and the final volume was 200 μl per well in 96-well polystyrene microtiter plates. In the biofilm stimulation experiments, TSB with 50 μg/ml AGE-BSA, TSB with 50 μg/ml BSA, or TSB with 40 mmol/l of glucose were used to incubate the biofilm, respectively. Following incubation at 37°C for 24 h, the plates were washed with phosphate buffered saline (PBS) three times to remove the unattached bacteria. Biofilms were then heat fixed at 60°C for 120 min, subsequently stained with 0.1% CV for 5 min at room temperature. Then, the CV was aspirated out and the plates rinsed with cold running water. Biofilms were dried at 35°C for 120 min, and then, photographs were taken with digital cameras (D-LUX7, Leica Inc., VA, United States). After this, the stained biofilm was resolubilized in 200 μl of 95% ethanol, and optical density (OD) was taken at 570 nm (Multiskan FC, Thermo Scientific Inc., MA, United States) to evaluate biofilm formation ability. *S. aureus* ATCC 25923 and *S. aureus* ATCC 29213 were used as positive and negative controls (ODc) for the biofilm formation and TSB broth as the blank control, respectively. All experiments were performed in triplicate.

### Live/Dead Analysis of Colony Biofilms by Confocal Microscopy

Overnight cultures of *S. aureus* grown in TSB were adjusted with TSB, TSB with 40 mmol/l glucose, TSB with 50 μg/ml AGE-BSA, or TSB with 50 μg/ml BSA, respectively, to an OD_600_ of 0.05. Three aliquots were inoculated in glass bottom culture dish for confocal laser scanning microscopy (CLSM) (MatTek, Ashland, OR, United States). After static culture at 37°C for 24 h, the biofilms were washed with PBS and stained using the LIVE/DEAD BacLight Bacterial Viability kit, then incubated for 15 min in the dark, and fixed with 4% paraformaldehyde. After adding PBS, the biofilms were visualized with a Zeiss confocal laser-scanning microscope (Zeiss LSM 800 with airy scan, Germany). The stain kit was the mixture of the SYTO9 and propidium iodide (PI) stains. SYTO9 was excited at 485 nm, and its emission was monitored at 498 nm; PI was excited at 535 nm, and emission was at 617 nm. With the staining, bacteria with damaged membranes exhibited red fluorescence, whereas bacteria with intact cell membranes displayed green fluorescence. In all cases, 0.8-μm optical sections of the biofilm were collected, the stacks of images were analyzed, and the biofilm parameters were determined using the Zen 2.3 software. The images of two positions randomly selected from two independent samples are analyzed.

### Biochemical Composition of the Biofilms Matrix

Biofilm formation was induced as described above. All wells were washed with PBS and treated for 1 h at 37°C, either with a solution of 100 μg/ml DNase I to disrupt the eDNA, or with 10 mM sodium metaperiodate to disrupt the extracellular polysaccharides, or with 100 μg/ml proteinase K to disrupt the biofilm extracellular protein (Dakheel et al., 2016). Subsequently, the biofilms were washed, fixed, and stained with CV as described above and the OD570 measured.

### Extraction and Quantification of eDNA

Extraction and quantification of eDNA was performed according to a previous study with some modifications (Rice et al., 2007). Briefly, bacteria were cultured in six-well plates at 37°C for 24 h. After that, the plates were chilled at 4°C for 1 h, and 1 μl of 0.5 M ethylenediaminetetraacetic acid (EDTA) was added to each well. After discarding the supernatants, the biofilms were resuspended in 400 μl of RB buffer and transferred into precooling tubes. After centrifugation at 4°C and 18,000 × *g* for 5 min, 200 μl of supernatant was transferred to a new chilled tube containing 300 μl of Tris–EDTA (TE) buffer. Proteins were removed first with phenol/chloroform/isoamyl alcohol (25:24:1) of equal volume and then with chloroform/isoamyl alcohol (24:1). Three volumes of ice-cold 100% ethanol were added to precipitate DNA. After centrifugation at 18,000 × *g* at 4°C for 20 min, the precipitate was washed with ice-cold 70% ethanol, air-dried, and dissolved in 20 μl of TE buffer. eDNA was quantified using a NanoDrop One^*c*^ spectrophotometer (Thermo Fisher, Waltham, MA, United States). The nanogram of eDNA per biomass of each biofilm was calculated by dividing the eDNA (ng) by its average OD600 value. All experiments were repeated in triplicate.

### RNA Extraction From *S. aureus*

*Staphylococcus aureus* biofilms were cultured in 75-cm^2^ polystyrene cell culture flasks (Corning, NY, United States) at 37°C statically for 24 h in TSB with 50 μg/ml AGE-BSA or TSB with 50 μg/ml BSA. The supernatants were removed after static incubation for 24 h, and the biofilms were scraped down using a cell scraper and then transferred to a new Eppendorf (EP) tube. The biofilms were disrupted with 40 μl of 1 mg/ml lysostaphin and 20 μl of 50 mg/ml lysozyme. Finally, the lysates were treated with Trizol Reagent^®^ according to the manufacturer’s protocol, and bacterial RNA was extracted.

### Real-Time Quantitative Reverse Transcription PCR

Bacterial RNA at 24 h biofilms was obtained as described above. cDNA synthesis was performed with the PrimeScript TM RT Master Mix (Perfect Real Time) according to the reagent instructions. Quantitative reverse transcription PCR (qRT-PCR) was performed using the SYBR Premix Ex Taq (Perfect Real Time) and Roche Light Cycler 480 II (Roche, Basel, Switzerland). The transcript copy number was determined using the Light Cycler 480 II system software, and the 16sRNA gene was used to normalize data. The 2^–ΔΔ*Ct*^ value represents the difference in threshold cycle (Ct) between the target and control (16sRNA) genes treated with BSA and AGE-BSA.

### Statistical Analysis

Results are reported as mean ± standard deviation (SD). Statistical analysis was performed with SPSS v20.0 (IBM Corp., New York, NY, United States). All experiments were repeated at least three times. Between-group comparisons were made using Student’s *t*-test. Multiple comparisons were carried out using one-way analysis of variance (ANOVA). Spearman correlation analysis was used to analyze the statistical significance between HbA1c (%) and biofilm formation capability (absorbance at 570 nm after CV staining). A two-tailed *p* < 0.05 was considered significant.

## Results

### *S. aureus* Isolated From Diabetic Chronic Skin Wounds Had Stronger Biofilm Formation Ability

**FIGURE 1:**
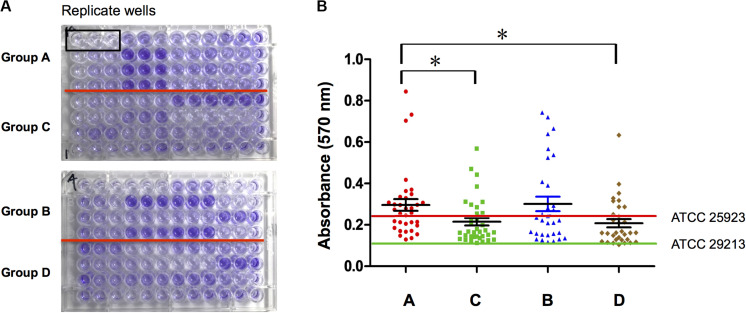
*S. aureus* isolated from diabetic chronic skin wound had stronger biofilm formation ability. **(A)** Ninety-six-well plates were stained with crystal violet and photographed: group A, isolates from diabetic foot wounds (>4 weeks); group B, isolates from the other chronic skin wounds of diabetic patients (>4 weeks); group C, isolates from chronic wounds of non-diabetic patients (>4 weeks); group D, isolates from acute wounds (<2 weeks); three biological replicates for each assay. **(B)** Determination of biofilm formation ability by semiquantitative measurement of absorbance at 570 nm after crystal violet staining (ATCC25923, the positive control of *S. aureus* biofilm; ATCC29213, the negative control of *S. aureus* biofilm). Compared with group A, *P < 0.05.

Biofilm formation ability of *S. aureus* isolates from four types of wounds was evaluated as mentioned in Section “Patients and Sample Collection” and displayed obviously differences. *S. aureus* isolates from diabetic patients were more prone to form biofilms than those from the non-diabetics, both in acute and chronic wounds. In the diabetic group, there was no difference in biofilm formation capacity of the strains from foot ulcers or other kinds of skin wounds, which meant that biofilm formation was universal in diabetes chronic wound infection, not limited to DFI (Figure 1). In order to investigate the possible reason for biofilm formation, we analyzed and compared the clinical index of the DB+ and DB- patients. Unexpectedly, there were few differences in the clinical index between the two groups, including blood glucose levels. However, the duration of diabetes and HbA1c were obviously higher in the DB+ than in the DB- ones (Table 2). Additionally, there was a positive correlation with HbA1c (%) and biofilm formation capability (absorbance at 570 nm after CV staining) (rs = 0.763, *P* < 0.01).

**TABLE 2:** Demographic and clinical characteristics of 65 diabetic foot infection patients.

	**Biofilm positive**	**Biofilm negative**
	**(*n* = 35)**	**(*n* = 30)**
Cases in group A/B (*n*)	20/15	14/16
Age (years)	52.5 ± 10.3	53.3 ± 7.2
Gender (Male/female)	16/18	14/17
Duration of DM (years)^a^	8.2 ± 4.2	5.9 ± 2.5
FBS (mmol/L)	9.4 ± 3.8	9.6 ± 4.0
HbA1c (%)^a^	10.1 ± 2.4	6.9 ± 2.8
TG (mmol/L)	4.3 ± 1.4	4.2 ± 1.0
TC (mmol/L)	5.4 ± 1.6	5.6 ± 1.2
LDL-C (mmol/L)	3.9 ± 0.4	4.1 ± 0.5

*DM, diabetes mellitus; FBS, fasting blood glucose; TC, total cholesterol; TG, total glyceride; LDL-C, LDL cholesterol; ^*a*^*i* < 0.05.*

### AGEs Enhanced Biofilm Formation of *S. aureus* Strains

**FIGURE 2:**
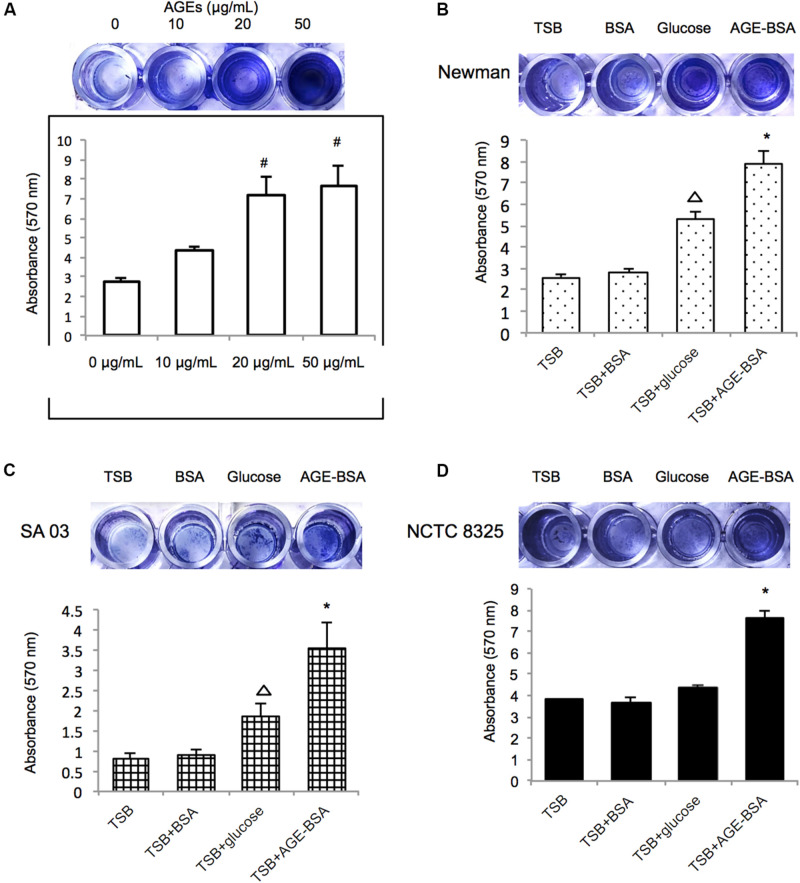
Advanced glycation end products (AGEs) enhance biofilm formation of *S. aureus* strains. **(A)** Biofilm-forming capability of *S. aureus* with varying AGE doses. Photograph of *S. aureus* Newman strain grown 24 h at different AGEs concentration after crystal violet staining, and the absorbance was measured at 570 nm (OD570). Three biological replicates for each assay. Photograph of *S. aureus* strain **(B)** Newman, **(C)** SA03, and **(D)** NCTC8325 grown 24 h at 50 μg/ml bovine serum albumin (BSA) group (control group), 40 mmol/l glucose, and 50 μg/ml AGEs-BSA group after crystal violet staining. The absorbance of different *S. aureus* strains was measured at 570 nm (OD570). #compared with 0 μg/ml, *P* < 0.05; △ compared with trypsin soybean broth (TSB) group, *P* < 0.05; ^∗^compared with TSB-BSA group, *P* < 0.05.

Initially, the Newman strain was chosen for treatment with different concentrations of AGE-BSA to establish the biofilm (Figure 2A). The biofilms of the strain grown in the presence of 20 and 50 μg/ml of AGE-BSA showed a significant increase and demonstrated concentration-dependent growth. Then, NCTC8325 and methicillin-resistant *S. aureus* (MRSA) clinical isolate SA03 were added to determine the effect of 50 μg/ml AGEs-BSA on biofilm formation because different strains could respond differently to AGEs-BSA. Both the MSSA and the MRSA strains showed increased biofilm production at 24 h after exposure in 50 μg/ml AGE-BSA (Figures 2B–D). The values of AGEs-biofilm induction ranged from 3.52 ± 0.98 (SA03) to 7.87 ± 1.41 (Newman) for the strains studied. Values of the biomass relative to the strain treated with BSA were as follows: NCTC8325 (1.85-fold), Newman (3.07-fold), and SA03 (4.13-fold).

**FIGURE 3:**
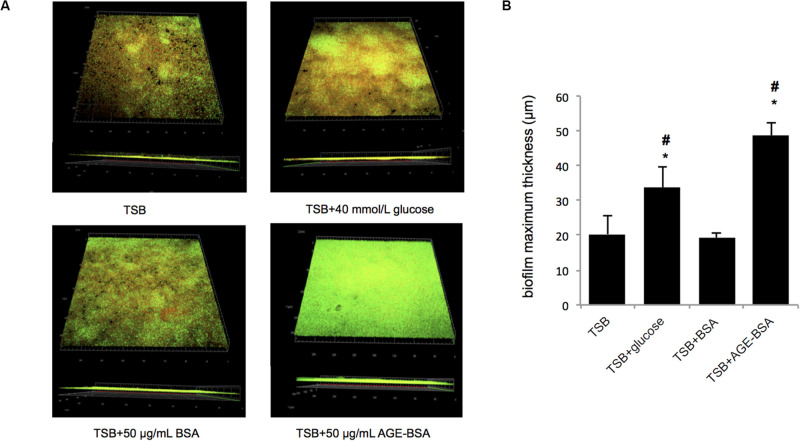
Biofilm structures and biomass of *S. aureus* strain Newman cultured in trypsin soybean broth (TSB) with 50 μg/ml advanced glycation end product (AGE)-bovine serum albumin (BSA) and other control, measured by confocal laser scanning microscopy (CLSM). **(A)** Live/Dead staining images. The viable bacteria were stained by SYTO9 (green) and PI stained the dead bacteria (red). The images were showed after merging (green, red, and yellow). **(B)** Comparison of biofilm thickness (*P < 0.05 compared with TSB group; #*P* < 0.05 compared with TSB + BSA group).

Furthermore, the biofilms formed by the Newman strain in the presence of AGE, AGE-BSA, and glucose were stained with the LIVE/DEAD BacLight Bacterial Viability kit and visualized by CLSM (Figure 3A). The ZEN 2.3 system analyses of images established that exposure to 50 μg/ml AGEs-BSA provoked the increment in the biofilm maximum thickness (48.67 ± 3.51 μm) when compared with the BSA control (maximum thickness, 19.33 ± 1.53 μm), and the glucose control (maximum thickness, 33.67 ± 6.03 μm) (Figure 3B). Additionally, the growth curve showed that AGEs had no effect on the growth of planktonic *S. aureus* (Supplementary Figure S1).

### AGEs Increased Biofilm Formation of *S. aureus* Strains Mainly via Increasing eDNA Release

**FIGURE 4:**
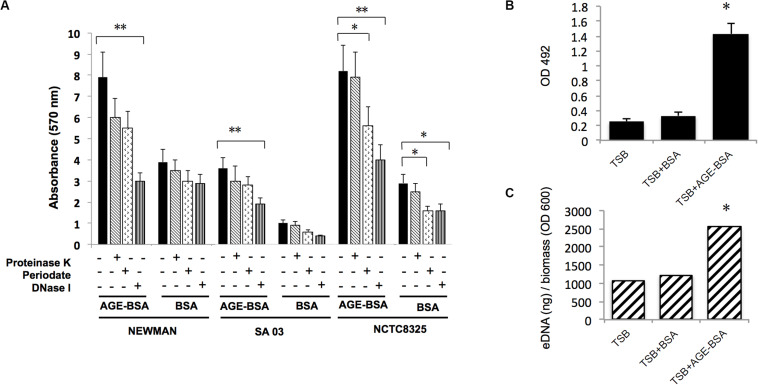
Advanced glycation end products (AGEs) increased biofilm formation of *S. aureus* strains mainly via increasing eDNA release. **(A)** Dissociation (DNase I, proteinase K, and sodium periodate) treatment experiment. The biofilm formation induced by AGE-bovine serum albumin (BSA) was significantly reduced after coincubation with DNase I compared with Tris buffer (20 mM, pH 7.5), whereas biofilm formation in control was not changed. *P < 0.05. **(B)** eDNA of Newman strain was extracted and determined. **(C)** eDNA per biomass of each biofilm was calculated via dividing the eDNA (ng) by its average OD600 value. Biofilms induced by AGE-BSA contained more eDNA than the control. *P < 0.05; **P < 0.01.

Three dissociations (DNase I, proteinase K, and sodium periodate) were used to treat stable biofilms induced by AGEs to evaluate the main components in the biofilm. The results (Figure 4A) showed that the biofilm induced by AGE-BSA of three *S. aureus* strains was obviously disrupted by DNase I, suggesting that AGEs induce the formation of *S. aureus* biofilm mainly by increasing eDNA. Then, eDNA extraction and quantification was used to confirm this result (Figures 4B,C).

### AGEs Induced eDNA Release by Positively Affecting *sigB* Transcription and Downregulating *lrgA* Expression

**FIGURE 5:**
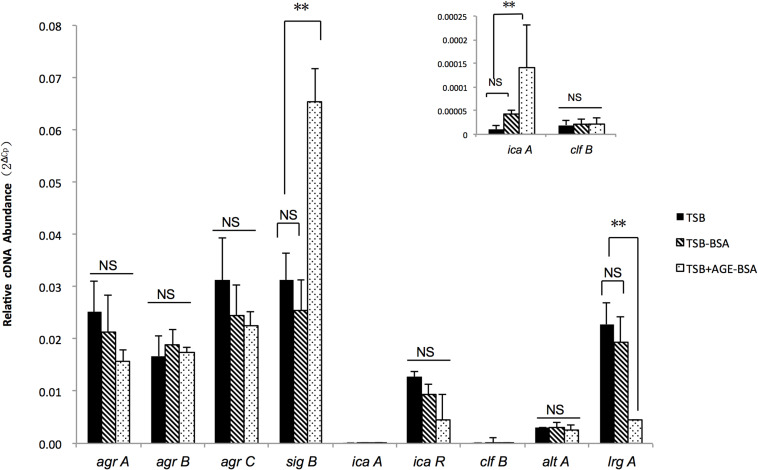
Reverse transcription quantitative PCR (RT-qPCR) detection of the expression level of *agrA*, *agrB*, *agrC*, *sigB*, *icaA*, *icaR*, *cifB*, *altA*, and *lrgA* genes in Newman strain cultured in trypsin soybean broth (TSB), TSB with BSA, or AGE-BSA. The *sigB*, *lrgA*, and *icaA* expression were changed in AGE-BSA group. **P < 0.01; NS, not significant.

**FIGURE 6:**
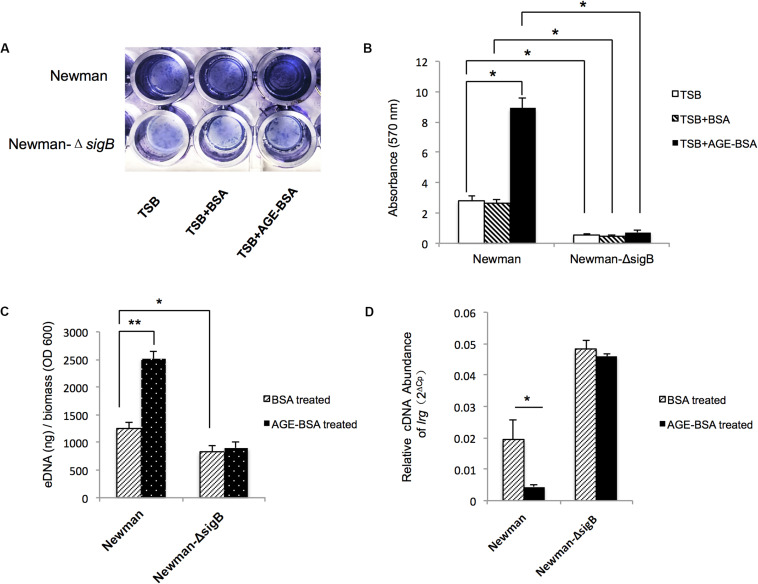
Advanced glycation end products (AGEs) induced e-DNA release by positively affected *sigB* transcription and downregulated *lrg A* expression. **(A)** Photograph of Newman strain and Newman-Δ*sigB* strain grown 24 h at TSB, TSB with 50 μg/ml BSA, and 50 μg/ml AGEs-BSA, after crystal violet staining. **(B)** The OD570 was detected (*n* = 3). *P < 0.05. **(C)** eDNA was extracted and determined. AGEs-BSA obviously increased the eDNA of the wild-type Newman, but had no effect on the eDNA of Newman-Δ*sigB* strain. *P < 0.05. **(D)** Reverse transcription quantitative PCR (RT-qPCR) detection of the expression level of *lrgA* gene in Newman and Newman-Δ*sigB* with or without AGE treatment. The *lrgA* transcription increased in Newman Δ*sigB* strain, and AGEs had no effect on the *lrg A* expression as that in Newman wild-type strain. *P < 0.05; **P < 0.01.

Two genes associated with eDNA release of *S. aureus*, *sigB* and *lrgA*, were identified and screened by qRT-PCR techniques (Figure 5). The transcription of *sigB* sharply increased in the AGE-BSA treatment group compared with the BSA group (*P* < 0.01), while the downstream genes *lrgA* were significantly decreased (*P* < 0.01). Besides, *icaA* appeared an obvious activation (*P* < 0.01) but still at a low expression level compared with other genes. This result suggested that the AGE-BSA promoted eDNA release possibly through the *sigB–lrg* pathway, in which *sigB* plays an important role. Sigma factor B is the key factor in the stress response system in *S. aureus*, closely related to drug resistance, toxin expression, and biofilm formation (Supa-Amornkul et al., 2019). To further investigate the role of *sigB* in this process, biofilms produced by the Newman *sigB-*deficient mutant (Newman-Δ*sigB*) grown in the presence of AGE-BSA or BSA were subjected to biofilm mass analysis. The biofilm mass was measured by CV staining and showed that the biofilm formation of Newman-Δ*sigB* was significantly decreased compared with that of Newman. AGE-BSA significantly improved the biofilm formation of Newman but had no effect on that of the *sigB-*deficient mutant (Figures 6A,B). Moreover, the eDNA amount per biofilm mass was decreased in the *sigB* mutant, and AGE-BSA had no obvious effect on the eDNA composition of the *sigB-*deficient mutant as it did on the wild type (1.09- versus 2.01-fold, *P* < 0.05) (Figure 6C). Therefore, *S. aureus* eDNA was affected by AGE-BSA in a *sigB*-dependent manner. qRT-PCR showed that *lrg A* transcription was increased in the *sigB-*deficient mutant and did not decrease as seen in the wild type after AGE-BSA administration (Figure 6D).

## Discussion

Biofilms of *S. aureus* are a predominant cause of persistent infections (Malachowa et al., 2015; Kalan and Brennan, 2019) and present a significant challenge for clinical doctors who treat patients suffering from DFI (Malik et al., 2013). Since there is still no effective treatment, prevention could be a new direction and strategy to deal with biofilms.

Bacteria colonization and biofilm formation include adherence, formation of an extracellular matrix (ECM), and dissociation. The alteration from planktonic phenotype to colonizing biofilm needs an “external signal.” Except for the signaling compounds of the bacteria themselves, i.e., quorum signals, a large number of host environmental signals participated in regulating biofilm formation, maturation, and dissociation. These signals include physical and chemical factors such as nutrients, pH, and the temperature of the host (Krishna and Miller, 2012; Santoro et al., 2015; Chen et al., 2018). Due to multiple chronic metabolism disorders, diabetics provide a “unique” host environment for the colonization of infectious bacteria, which in turn affects the bacteria’s adhesion, invasion, and toxin, even the resistance to drugs (Dunyach-Remy et al., 2016). Previous studies have demonstrated that diabetic patients had more potential to colonize biofilm bacteria. Gulia et al. (2018) found that diabetic saliva has more potential to form a biofilm of *Candida albicans* and *Streptococcus* mutans than non-diabetic saliva. Longo et al. (2018) analyzed the subgingival microbiome in diabetic patients and found that subgingival biofilm composition was significantly different in type 2 diabetes mellitus (DM) patients. These studies mainly focused on oral infections such as periodontitis; few studies have addressed skin and soft tissue infection, although biofilm infection plays an important role in chronic wounds (Malone et al., 2017; Wu et al., 2019). This study presented that *S. aureus* strains isolated from diabetic chronic wounds are more prone to form biofilms than the non-diabetic chronic wounds, consistent with previous studies.

Hyperglycemia and high glycation products are the most striking features of diabetes (Huijberts et al., 2008). In many *in vitro* studies, glucose was found to induce biofilm formation of bacteria (Rode et al., 2007; Waldrop et al., 2014; Kyoui et al., 2016; She et al., 2019), it is not enough to explain the difference in severity of infection and antibiotic therapy effects between patients with similar blood sugar levels. As this study showed, we did not find differences in blood glucose levels between DB+ and DB- patients. This might be attributed to large fluctuations in glycemia levels, unstable tissues glucose concentration, and more active glycemia control of DFI patients. Furthermore, HbA1c were higher in biofilm-positive diabetics, consistent with previous research that showed that HbA1c ≥ 8% is associated with increased subgingival biofilm in subjects with type 2 DM (Miranda et al., 2017). In this study, we found that HbA1c was positively correlated with biofilm formation ability of *S. aureus*. As a glycosylation product in peripheral circulating blood, high HbA1c represents poorer glycemic control but cannot directly reflect the tissue glucose environment. As a reflection of hyperglycemia in tissues, AGEs have recently received more and more attention in research addressing age-related diseases, diabetes, and complications stemming from diabetes (Mitsuhashi et al., 1993; Stensen et al., 2014; Vlassara and Uribarri, 2014; Wautier et al., 2014). Our previous studies showed that compared with continuous hyperglycemia, AGEs have a stronger influence on apoptosis, migration, and regeneration of wound cells, which are considered to be major factors in the difficult process of diabetic wound healing (Zhang et al., 2016). Because the synthesizing process is an instantaneous and irreversible reaction, AGEs continue to accumulate in tissues with age and high glycemia duration (Huebschmann et al., 2006; Costa et al., 2017). In this study, another risk factor besides HbA1c was DM duration, suggesting that high AGEs in diabetic foot ulcer (DFU) tissue could play a role in the formation of biofilm of *S. aureus.* In summary, we speculated that AGEs are major factors of biofilm formation in diabetics. Further experiments in this study confirmed this hypothesis. The result showed that AGE exposure significantly increased biofilm formation in three *S. aureus* strains, even higher than glucose, whether in biofilm mass, density, or amount of living bacteria present, whose exact mechanism needs more investigation.

*Staphylococcus aureus* biofilms consist of complex ECMs, including PIA, eDNA, and proteins (Hall-Stoodley et al., 2004). Here, we found that AGEs induced biofilm formation mainly by increasing eDNA mass. As a major structural component of many different microbial biofilms, the importance of eDNA was first reported in *Pseudomonas aeruginosa* (Whitchurch et al., 2002). eDNA is released via autolysis in a suicidal or fratricidal manner and/or active release through membrane vesicles and nanofibers in biofilms. Considerable studies have confirmed the crucial role of eDNA in the formation, adhesion, and stability of bacterial biofilms in general (Barnes et al., 2012).

The regulatory network involved in eDNA formation is complicated. As a whole, holin/antiholin molecules (CidA and LrgAB) (Ranjit et al., 2011), other autolysins (Yycl) (Beltrame et al., 2015), two-component systems (Agr, ArlRS, LytSR, SaeRS, WalKR, and SrrAB) (Arciola et al., 2012), and global regulators (SigB, SarA and MgrA, and Rot) directly and/or indirectly regulate this process. As the predominant stress responses of several Gram-positive bacteria (van Schaik and Abee, 2005), the alternative Sigma factor B plays a crucial role in the regulation of gene expression in response to changes in environments and is an essential regulator of *S. aureus* biofilm formation. In this study, we found AGEs deregulated *sigB* transcription, independent of the two-component systems Agr and SarA, and further confirmed the essential role of *sigB* in AGE-induced biofilm formation using the *sigB* deletion mutant strain. It was reported that *sigB* gene-deleted mutants were unable to create biofilms in a CA-MRSA lineage USA300 (Kiedrowski et al., 2011); however, in the present study, we identified a *sigB* deletion mutation in backgrounds of Newman strain that could form biofilms of *S. aureus*, although the biofilm was thinner than the Newman wild type.

Sigma factor B regulates biofilm formation by regulating various genes implicated in the onset and maintenance of biofilms (Nicholas et al., 1999), including the *icaADBC* operon to affect PIA production, and other ica-independent genes such as *clfA*, *hla*, and *cid/lrg* to affect eDNA release (Supa-Amornkul et al., 2019). qRT-PCR revealed that *lrg* transcription decreased significantly with the increase in *sigB* after AGE treatment. *lrg* was proposed to encode an antiholin-like protein with an inhibitory effect on murein hydrolase activity to inhibit cell lysis and eDNA release (Ranjit et al., 2011). Although we found that AGEs reduced *lrg* transcription and increased eDNA release in the Newman strain, the exact mechanism need more investigation. As previously reported, *lrg* was negatively regulated by *sigB* and played a role in the *sigB*-mediated stress response (Rice et al., 2004). When analyzed for the role of *sigB* in *S. aureus* biofilm development, the Δ*sigB* mutant displayed increased transcription of *lrg* with the decrease in eDNA and biofilm mass. Remarkably, unlike the wild-type Newman strain, both the *lrg* transcription and eDNA synthesis appeared to have no significant changes in the *sigB* deletion mutation after the administration of AGEs. This implied that AGEs do not directly inhibit *lrg* transcription to increase biofilm synthesis, but do so through a *sigB–lrg*-eDNA-dependent pathway.

Taken together, our data demonstrate that the presence of AGEs, an important environmental factor in diabetic foot tissue, strongly promotes *S. aureus* biofilm production by increasing eDNA release. These effects are the consequence of increasing sigB transcription and the subsequent downregulation of its downstream gene, *lrgA*. Indeed, the increase in *S. aureus* biofilm production induced by AGEs may contribute to infection persistence in DFI. The focus of this study is to examine the role of unique host chemical signals in modulating bacterial phenotype expression, specifically that of biofilm formation. This study tried to provide a new strategy to prevent biofilm formation by changing the host environment, such as by decreasing AGE levels in DFI patients.

## Data Availability Statement

All datasets generated for this study are included in the article/Supplementary Material.

## Ethics Statement

Ethics approval for this study was granted by the Ethics Committee of Sun Yat-sen Memorial Hospital (Reference number: SYSEC-KY-KS-2020-003). The patients/participants provided their written informed consent to participate in this study.

## Author Contributions

All authors listed have made a substantial, direct and intellectual contribution to the work, and approved it for publication.

## Conflict of Interest

The authors declare that the research was conducted in the absence of any commercial or financial relationships that could be construed as a potential conflict of interest.

## Supplementary Material

FIGURE S1 Growth curves of *S. aureus* Newman strain with different incubation.

DATA SHEET S1 Primer information.
