# Supplementary figures and images for: Advanced Glycation End Products Enhance Biofilm Formation by Promoting Extracellular DNA Release Through sigB Upregulation in Staphylococcus aureus
Source: Front Microbiol. 2020 Jul 14;11:1479. doi: 10.3389/fmicb.2020.01479 (PMC7381169; doi:10.3389/fmicb.2020.01479)

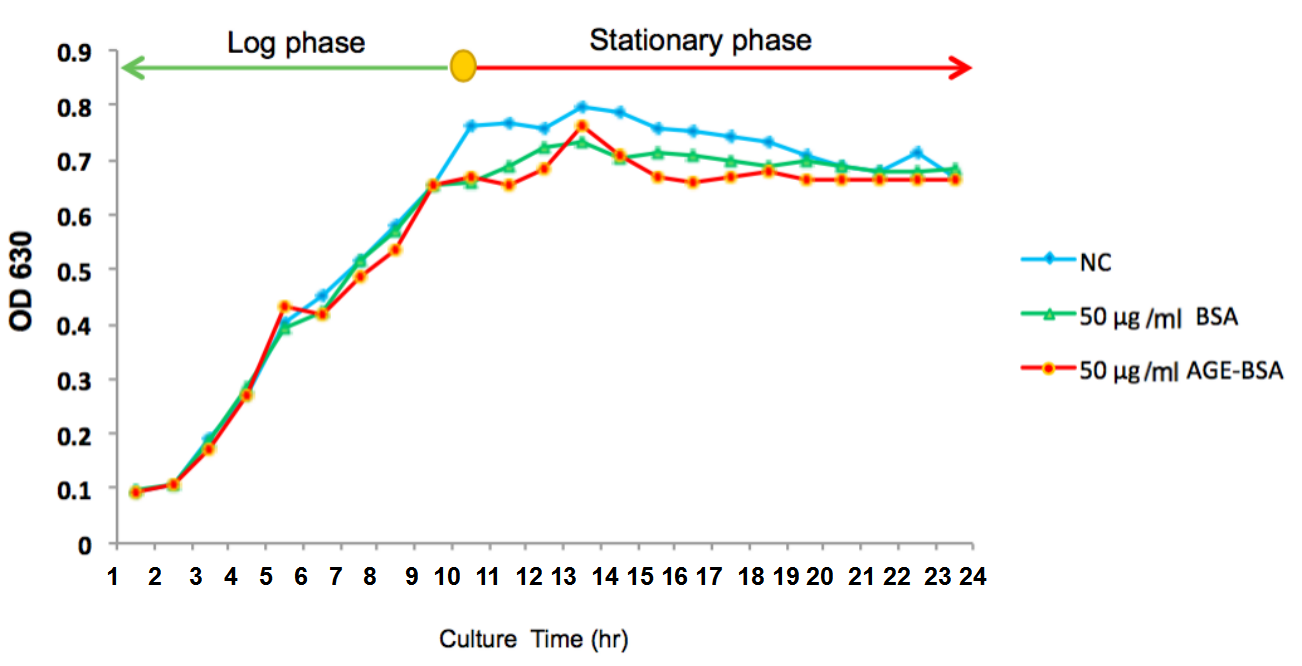

Supplement: FIGURE S1 — Growth curves of S. aureus Newman strain with different incubation. [file Image_1.TIF]

Supplementary Material

Supplementary Material 2: Primer information


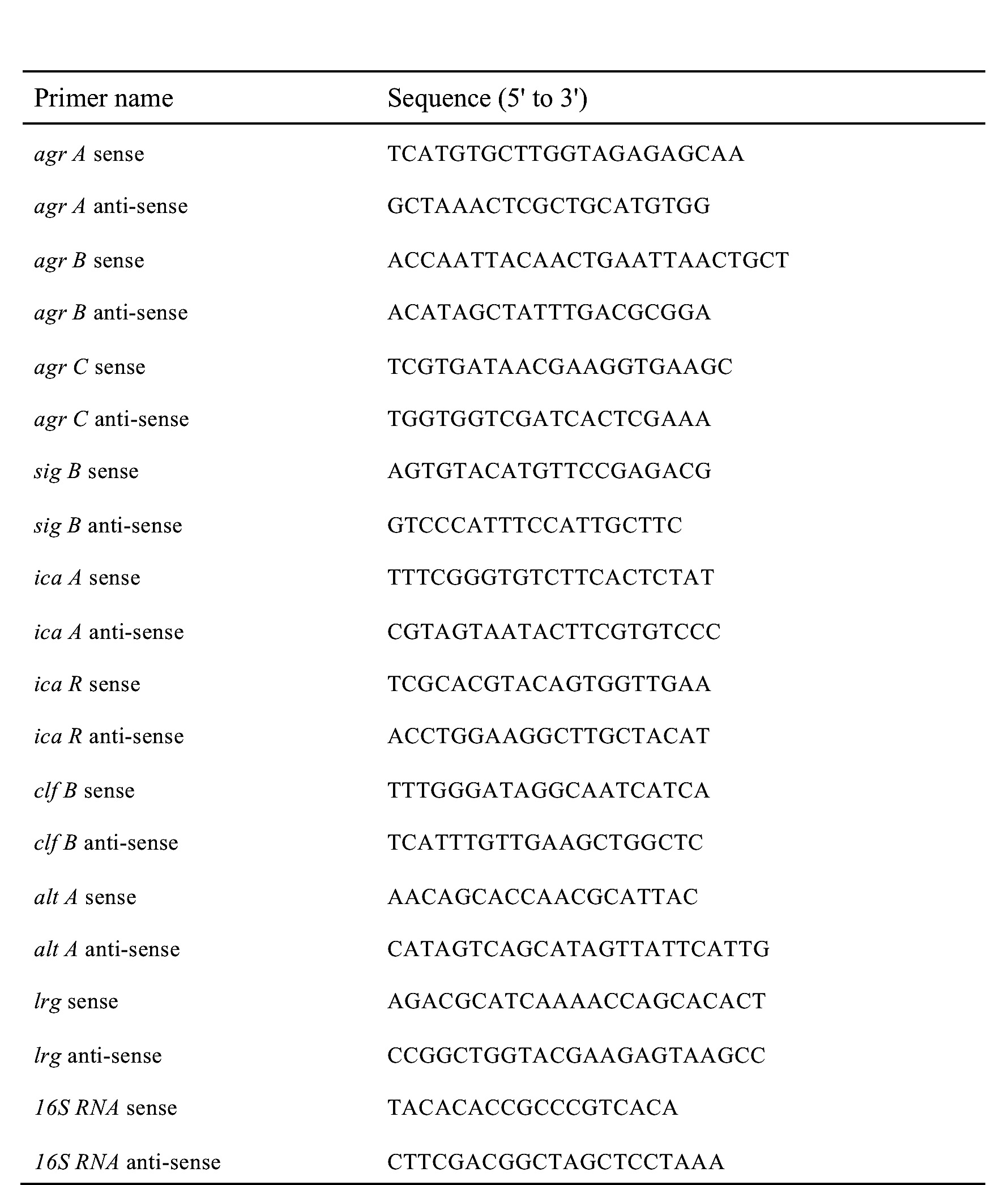

Supplement: DATA SHEET S1 — Primer information. [file Data_Sheet_1.docx]
